# Cholesterol associated genetic risk score and acute coronary syndrome in Czech males

**DOI:** 10.1007/s11033-023-09128-3

**Published:** 2024-01-22

**Authors:** Jaroslav A. Hubacek, Vera Adamkova, Vera Lanska, Vladimir Staněk, Jolana Mrázková, Marie Gebauerová, Jiri Kettner, Josef Kautzner, Jan Pitha

**Affiliations:** 1 Experimental Medicine Centre, Institute for Clinical and Experimental Medicine, IKEM-CEM-LMG, Videnska 1958/9, 140 21 Prague 4, Czech Republic; 2 3rd Department of Internal Medicine, 1st Faculty of Medicine, Charles University, Prague, Czech Republic; 3 Preventive Cardiology Centre, Institute for Clinical and Experimental Medicine, Prague, Czech Republic; 4 Information Technology Division, Institute for Clinical and Experimental Medicine, Prague, Czech Republic; 5 Cardiac Centre, Institute for Clinical and Experimental Medicine, Prague, Czech Republic

**Keywords:** Cholesterol, Acute coronary syndrome, Polymorphism, Risk estimation

## Abstract

**Background:**

Despite a general decline in mean levels across populations, LDL-cholesterol levels remain a major risk factor for acute coronary syndrome (ACS). The *APOB*, *LDL-R*, *CILP*, and *SORT-1* genes have been shown to contain variants that have significant effects on plasma cholesterol levels.

**Methods and results:**

We examined polymorphisms within these genes in 1191 controls and 929 patients with ACS. Only rs646776 within *SORT-1* was significantly associated with a risk of ACS (P < 0.05, AA vs. + G comparison; OR 1.21; 95% CI 1.01–1.45). With regard to genetic risk score (GRS), the presence of at least 7 alleles associated with elevated cholesterol levels was connected with increased risk (P < 0.01) of ACS (OR 1.26; 95% CI 1.06–1.52). Neither total mortality nor CVD mortality in ACS subjects (follow up—9.84 ± 3.82 years) was associated with the SNPs analysed or cholesterol-associated GRS.

**Conclusions:**

We conclude that, based on only a few potent SNPs known to affect plasma cholesterol, GRS has the potential to predict ACS risk, but not ACS associated mortality.

## Introduction

Increased plasma cholesterol (especially within the LDL fraction), together with smoking, obesity, diabetes, and hypertension, is considered a major contributor to atherosclerotic cardiovascular disease (ACVD) and subsequent acute coronary syndrome (ACS).

Genetic background is an important factor when determining the final values of plasma lipids. Rare monogenic causes of high plasma cholesterol levels have been documented [1]. However, in the majority of subjects, a wide list of genes and variants that exert relatively small but measurable effects can also influence, in addition to unhealthy lifestyles, final plasma lipid concentrations.

The potential association between plasma cholesterol concentration and myocardial infarction was first documented many decades ago [2, 3]. On the one hand, most of the studies that have employed univariable Mendelian randomisation analysis highlight a causality between LDL-cholesterol values and cardiovascular disease [4, 5]. On the other hand, detailed and extensive studies that have utilised the multivariable MR-Egger method have categorically failed to confirm such an association [6, 7]. In fact, several studies suggest that examination of APOB plasma levels is sufficient for risk estimation and, furthermore, that the inclusion of cholesterol values does not further improve risk prediction [6, 8].

For our study, we selected 4 SNPs with a proven and highly significant impact on plasma total cholesterol and LDL-cholesterol in the general Czech population [9]. The first, rs693, is located within *APOB* (apolipoprotein B, major protein component of LDL particles, OMIM acc. No. 107730); the second, rs16996148, is located at the *CILP2/PBX4* loci (cartilage intermediate layer protein 2; OMIM acc. No. 612419 and pre-β-cell leukaemia transcription factor 4; OMIM acc. No. 608127); the third, rs6511720, is located within *LDL-R* (LDL-receptor, a key protein involved in cholesterol catabolism; OMIM acc. No. 606945); and the fourth, rs646776, is located within the *SORT-1* gene (involved in hepatic transport of lipoproteins and arterial calcification; OMIM acc. No. 602458).

The respective importance of the above variants in determining plasma lipid values has been established by independent genome-wide association studies (GWAs) [10–13] and widely confirmed in different populations. Interestingly, these four SNPs represent just a minority of SNPs (4 out of 26, Hubacek, unpublished results), with confirmed strong effect in Czech general population. Given the polygenic background of hypercholesterolaemia, a genetic risk score (GRS) can be established from these four variants. In different ways, GRSs reflect the simultaneous effect of several individual polymorphisms and are, therefore, believed to be better predictors of disease risk [14, 15] than determinations based on individual genetic variants.

Noteworthy, in observation study protocols, we [16] and others (for example [17, 18]) have recently failed to prove, that increased levels of plasma cholesterol are undoubtedly associated with acute coronary syndrome in different population. This could be partially influenced by the fact, that plasma lipid values generally improved due to the last decades [19] as well as by the fact, that plasma cholesterol is slightly going down after acute coronary syndrome attack [20, 21]. Considering the effect of these variants on plasma cholesterol concentrations, we hypothesized that, if plasma cholesterol remains major traditional risk factor of ACS, individual SNPs and the establishment of a simple cholesterol-determining GRS would reflect an increased risk of ACS in Czech males.

## Materials and methods

### Subjects

This study included 929 male patients with ACS under the age of 65, consecutively enrolled between April 2006 and February 2015 according to a protocol used at the Cardiology Unit of the Institute for Clinical and Experimental Medicine, Prague (described in detail previously) [22, 23]. Data on mortality [24] were obtained from the Institute of Health, Informatics and Statistics (Ministry of Health of the Czech Republic), where all death certificates are analysed. Reached mean follow-up has been 9.84 ± 3.82 years.

For the control group, 1 191 males aged 25–64 years, all of whom participated in the Czech branch of the post-MONICA study [25]), were selected according to the WHO protocol [26], representing a 1% general population sample from 9 different Czech districts.

To screen for traditional cardiovascular risk factors (cholesterol values, smoking, obesity, hypertension, and diabetes), we used examination procedures described in detail previously [22, 23].

All study participants were self-reported Caucasians.

### Genetic analyses

DNA has been isolated from whole EDTA blood samples as described by Miller et al. [27]. Variants within the *APOB*, *CILP2/PBX4*, *LDL-R* and *SORT-1* loci were genotyped by PCR–RFLP as described in details before [28]. Briefly, DNA fragment obtaining *APOB* rs693 variant has been amplified using the 5ʹ aga gga aac caa ggc cac agt tgc and 5ʹ tac att cgg tct cgt gta tct tct oligonucleotides and restriction enzyme XhoI was used to distinguish allele C (uncut PCR product) and T (restriction fragments 110 bp + 26 bp). Oligonucleotides 5ʹ atc cag cta ttt ggg agc agt gtc ctg g and 5ʹ aag gtc tgg tct ctg gaa aac aga ag amplified gene part of *SORT-1*; restriction enzyme Hin1II produced fragments of 107 bp + 32 bp (allele G) with uncut product of 139 bp representing the allele A. *LDL-R* has been genotyped by oligonucleotides 5ʹ acc ggg gat gat gat gat tgc and 5ʹ ttg cct aag act tca tta aca ttt g. Alleles G (PCR product of 132 bp) and T (fragments of 106 bp + 26 bp) were distinguished after treatment with enzyme DpnI. The last polymorphism (*CILP2/PBX4* loci) has been genotyped with oligonucleotides 5ʹ tgg ctc ttg tcc act ggc cac atc ccc and 5ʹ ttc tcc cat gcc tcc agg ccc cca ag. Restriction enzyme Hin1II produced fragments of 82 bp + 54 bp (allele T) and uncut product of 137 bp is characteristic for the A allele.

### Statistical analysis

Chi-square tests and odds ratios (95% CI) were calculated using the freely available Social Science Statistics statistical software package (https://www.socscistatistics.com; accessed February 2022); all procedures used are fully compatible with SPSS software. Cases with fewer than 5 subjects in one category were pooled and analysed together with heterozygotes. A P-value below 0.05 was considered significant.

For calculation of the genetic risk score (GRS), only subjects possessing all 4 SNPs of interest were included (N = 1095 for controls and N = 886 for patients). An unweighted gene score was created for each individual, where subjects received one point for each cholesterol-increasing allele based on associations with plasma cholesterol levels in the general population sample. Due to the low numbers in groups on the opposite end of the score distribution curves, pooling was performed to create GRS sub-groups of “4 or fewer risk alleles” and “7 or more risk alleles”.

As examined SNPs have not been associated with any of the traditional risk factors (prevalence of hypertension, diabetes, smoking status or BMI values) no adjustments were performed.

## Results

### General characteristics of subjects

The general characteristics of ACS patients and controls are summarized in Table 1. As expected, there were more smokers, diabetics, and hypertensive subjects in the ACS group.

**Table 1:** General characteristics of examined subjects

N	Controls	ACS patients	P
	1 191	929	
Age (years)	49.0 ± 10.8	54.6 ± 8.0	0.01
BMI (kg/m^2^)	28.2 ± 4.0	28.6 ± 4.3	n.s.
Ever smokers (%)	58.7	84.9	0.00001
Hypertension (%)	40.7	50.6	0.00001
Diabetes (%)	8.9	18.0	0.00001
Total cholesterol (mmol/L)	5.75 ± 1.06	4.83 ± 1.12	0.001
LDL-cholesterol (mmol/L)	3.56 ± 0.99	3.58 ± 2.51	n.s.
Triglycerides (mmol/L)	1.97 ± 1.28	2.05 ± 1.50	n.s.
Follow-up (years)	n.a.	9.84 ± 3.82	n.a.

### Effects of individual SNPs

In three out of four of the examined SNPs (within the *APOB*, *LDL-R*, and *CILP/PBX4* loci), overall there was no association between ACS and individual genotypes associated with increased plasma cholesterol values. However, we did detect a slight difference in the case of *SORT-1* SNP. AA homozygotes, which have been strongly associated with high plasma levels of total and LDL-cholesterol (P < 0.005) and decreased levels of HDL-cholesterol (P < 0.005) (for more details, see [9]), were slightly more frequent in ACS subjects (64.5% vs. 60.0%; P = 0.03 for AA vs. + G comparison; OR 1.21; 95% CI 1.01–1.45) (Table 2).

**Table 2:** Genotype distributions of individual SNPs in ACS patients and controls

rs693	Controls		Patients		OR	P	P*
APOB	N	%	N	%	Crude		
CC	327	28.0	239	26.0	1.00		0.90^&^
CT	554	47.4	452	49.2	1.11 (0.91–1.37)	0.30	0.58^#^
**TT**	287	24.6	228	24.8	1.08 (0.85–1.38)	0.50	0.31^§^

rs16996148	Controls		Patients		OR	P	P*
CILP/PBX4	N	%	N	%	Crude		
**GG**	979	82.5	772	84.4	1.00		0.55^&^
GT	193	16.3	134	14.6	0.88 (0.69–1.12)	0.30	0.49^#^
TT	15	1.3	9	1.0	0.76 (0.33–1.75)	0.52	0.25^§^

rs6511720	Controls		Patients		OR	P	P*
LDL-R	N	%	N	%	Crude		
**GG**	982	82.7	747	74.3	1.00		0.11^&^
GT	194	16.3	136	15.4	0.92 (0.73–1.18)	0.50	0.22^#^
TT	11	0.9	3	0.3	0.36 (0.10–1.29)	0.10	0.34^§^

rs646776	Controls		Patients		OR	P	P*
SORT-1	N	%	N	%	Crude		
**AA**	688	60.0	579	64.5	1.00		0.07^&^
AG	394	34.4	283	31.5	0.85 (0.71–1.03)	0.10	0.05^#^
GG	65	5.6	35	3.9	0.63 (0.42–0.98)	0.04	0.04^§^

Genotypes associated with increased plasma levels of total and LDL-cholesterol are in bold. P* is calculated for dominant^&^, co-dominant^#^ and recessive^§^ models of comparisons

### Genetic risk score and risk of ACS

**Fig. 1:**
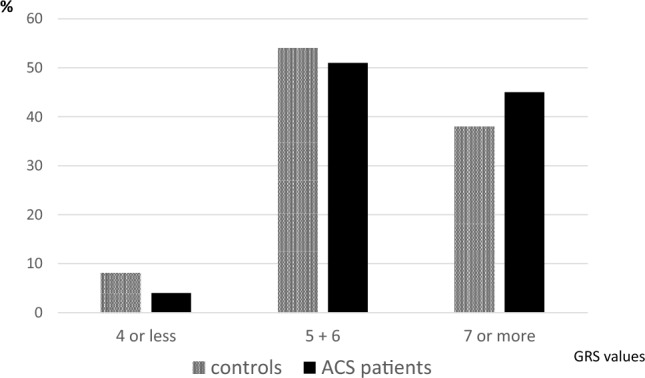
Distribution of genetic risk score in ACS patients and controls

In contrast, the results in respect of GRS were highly significant. As described previously in control subjects [9], total cholesterol and LDL cholesterol increase from those with the lowest to the highest score (P < 0.001 for linear trend). Carriers of 4 or fewer cholesterol-increasing alleles have a significantly lower (P < 0.005) risk of suffering from ACS (OR 0.55; 95% CI 0.37–0.82). Carriers of at least 7 risk alleles are at greater (P < 0.01) risk of ACS (OR 1.26; 95% CI 1.06–1.52) than others (Fig. 1).

### Long-term post-ACS mortality

The frequencies of individual SNPs were almost identical (all P-values above 0.44) between ACS survivors and non-survivors (Table 3). GRS was not associated with mortality in CVD subjects (not shown in detail).

**Table 3:** Genotype distributions of individual SNPs in ACS survivors and nonsurvivors

rs693	Survivors		Deceased		OR	P
APOB	N	%	N	%	Crude	
CC	192	26.1	47	25.8	1.00	
CT	362	49.1	90	49.5	1.01 (0.69–1.51)	0.94
**TT**	183	24.8	45	24.7	1.01 (0.63–1.59)	0.98

rs16996148	Survivors		Deceased		OR	P
CILP/PBX4	N	%	N	%	Crude	
**GG**	620	84.6	152	83.5	1.00	
GT	107	14.6	27	14.8	1.03 (0.65–1.63)	0.90
TT	6	0.8	3	1.6	n.a.	0.72*

rs6511720	Survivors		Deceased		OR	P
LDL-R	N	%	N	%	Crude	
**GG**	588	83.8	159	85.9	1.00	
GT	111	15.8	25	13.5	0.83 (0.52–1.33)	0.44
TT	2	0.3	1	0.6	n.a.	0.49*

rs646776	Survivors		Deceased		OR	P
SORT-1	N	%	N	%	Crude	
**AA**	462	64.2	117	66.1	1.00	
AG	227	31.5	56	31.6	0.97 (0.68–1.39)	0.89
GG	31	4.3	4	2.3	n.a.	0.63*

Genotypes associated with increased plasma levels of total and LDL-cholesterol are in bold

*Calculated for major homozygotes vs minor allele carriers

## Discussion

In our study, Mendelian randomisation (MR) analysis revealed a slight association between plasma cholesterol and increased risk of ACS. With regard to MR, genetic polymorphisms associated with selected biomarkers were used as proxies. Here, four genetic variants were strongly associated with the most frequently cited risk factor of cardiovascular disease (plasma LDL-C levels) and ACS prevalence was dependent (in a slight extent) on genetic risk score established from the four LDL-C associated SNPs.

The importance of individual SNPs in determining anthropometric and/or biochemical parameters is long acknowledged [29–31]. However, the vast majority of parameters are determined polygenously and the effect size of individual SNPs is relatively low (albeit functionally significant) and thus represents only minor part in determination of variability of parameters.

For a more nuanced interpretation of the simultaneous effect of several independent genetic variants, different types of genetic risk scores can be created. The respective influences of individual variants at different loci are generally converted into a single value in two ways [14, 15]: an unweighted genetic risk score is the simple sum of risk alleles present in each subject, whereas a weighted genetic risk score is calculated based on the effect size or OR/HR value of each participating SNP, meaning the final value can be time-dependent. Accordingly, absolute values will differ for identical subjects based on age at the time of examination and lifestyle at the time of blood collection.

The importance of GRS is underlined by recent opinions stating that GRS reflects lifetime exposure to risk factors (in the case of our study, to LDL-C levels) and that cumulative load is more precise in risk prediction [32].

Although original GWAs focused on the genetic determination of plasma lipids [10–13] typically include many thousands of subjects, these results are only useful for clinical purposes when replicated in each particular population. In fact, the effects of about one quarter of variants associated with LDL-cholesterol are not shared between subjects of different ancestry [33], while for triglycerides this “between population” transferability is even lower.

Thus, it is preferable to optimize GRSs for different populations, as not all variants exhibit the same effect in every population [34, 35]. GRS tailoring is especially necessary in cases where it is employed to predict diseases of affluence such as cardiovascular conditions, diabetes, and obesity. In these instances, a substantial proportion of risk is attributable to unhealthy lifestyle factors. The resulting potential inter-population differences can also be associated with gene-environment interactions. In such cases, the identical genetic variant can exert a variety of effects in different [14], or even in identical [36], populations according to environmental conditions.

Our previous analysis [9] clearly confirmed that all four GWAs-retrieved SNPs had a significant effect on plasma LDL-C levels in the Czech population. The effect size was between approximately 5% and 13% for individual SNPs. Both LDL-C and TC were higher by ~ 0.5 mmol/L for carriers of 8 risk alleles than for carriers of 4 or fewer risk alleles, a finding comparable with several previous studies. For example, one report found that a GRS established based on 23 SNPs was associated with TC levels between ~ 5.2 mmol/L (carriers of 11 or fewer cholesterol-raising alleles) and ~ 6.0 mmol/L for subjects with 18 or more alleles [37]. Shahid et al. found atherogenic blood lipids to have a significant positive association with GRS [38]. Their score, derived from 21 SNPs, revealed TC values ranging from ~ 4.7 mmol/L in carriers of 14 or fewer cholesterol-raising alleles to ~ 5.7 mmol/L in carriers with 21 or more risk alleles. An analysis of two UK biobank studies (WHII and BWHHS) [39] demonstrated differences between opposite GRS quintiles ranging from 0.6 to 0.9 mmol/L for both LDL-C and TC. Interestingly, the authors used different (albeit significantly overlapping) sets of SNPs to determine TC (20 SNPs) and LDL-C (22 SNPs). Finally, almost 8 500 SNPs were used to establish a GRS for White British UK Biobank participants, accounting for over 20% of the variance in LDL-C concentration [40].

As recently underlined GRS are important [41], but not all-explanatory [42] tools to predict medical events. Importantly—we believe, that the analysis of genetic predisposition to any noncommunicable disease needs to be (to be clinically useful) performed in time (at young age, probably at 25 years latest) and before the any onset of traditional risk factors. This was the secondary reason not to adjust our results for potential confounding factors. At this age, genetic predisposition could point on subjects under risk. Using the tools as intensive lifestyle intervention and more intensive and more focused screening programs, diseases onset could be, if not fully avoided, at least postponed to higher age categories.

Similar to screening for FH-causing mutations, screening for several common variants combined with a simultaneous/cumulative analysis of their effects can clearly identify potential patients at increased risk of hypercholesterolaemia and subsequent cardiovascular disease [43]. Nonetheless, it should be noted that the abovementioned studies significantly differed not only in terms of the number of SNPs but also in the method of selection.

There are uncertainties concerning the ideal number of variants to include in GRS calculations. A wide list of variants with only subtle effect sizes can in fact compromise the accuracy of results. The numbers of examined subjects used to identify risk allele/effect size are time-dependent and affected by potential selection bias. The smaller the cholesterol-raising effect size detected, the greater the chance of false-positive results. The actual effect across an entire population may be in fact zero or even negative. The “over-inclusion” of these variants in GRS calculations also results in a low cost–benefit. For example, Khera et al. [44] analysed an extreme 6.6 million SNPs to quantify CVD risk, where the top 5% of subjects with the highest GRS had “only” a 3.7-fold increased risk (nominally 17.5% of patients fell within this category compared to 5% within the rest of the population) of early onset myocardial infarction in comparison with other individuals. Shahid et al. [38] documented only a slightly lower OR (2.96) using a GRS based on a mere 21 SNPs.

Despite focusing on a different lifestyle-associated disease, namely type 2 diabetes mellitus, one study, which used results from the Estonian Biobank cohort, demonstrated that a GRS established from a maximum of 1000 SNPs is a better predictor of disease than a score established from a higher number of SNPs [45].

Two secondary outcomes of our study are noteworthy. Firstly, genotypes associated in the general population with increased plasma LDL-cholesterol values were more frequent than genotypes associated with lower plasma LDL-cholesterol values. Thus, it can be assumed that selection pressure during the ancient era of human development resulted in the “promotion” of these alleles. It is probable that one of the benefits of the increase in plasma cholesterol levels over time was to counteract infection [46]. The authors of a Dutch study have documented the advantage of higher total cholesterol in a historical context, finding that carriers of FH-causing mutations lived significantly longer until about the end of the nineteenth century than non-carriers [47].

Secondly, genotypes associated with increased plasma LDL-C values in the general population were only very slightly (and mostly non-significantly) over-represented in patients with ACVD compared to controls. Although we found a continuous increase in plasma LDL-C values in subjects along with a sequentially increasing number of risk alleles, only the subgroup comprising individuals with at least 7 risk/cholesterol-increasing alleles, i.e. in subjects with really high plasma cholesterol levels, were at increased risk of ACS. Nonetheless, although there was a trend toward higher GRS values in patients, the differences between patients and controls were more evident at the opposite end of the distribution curve, characterized by low GRS values.

In conclusion, our study indicates that genetic risk score, based on only a few individual SNPs, is a significant predictor of acute coronary syndrome in the Czech population even in cases where individual SNPs are associated with plasma cholesterol but not with increased risk of ACS per se. Importantly predisposition to low plasma cholesterol levels seems to be of greater importance than a predisposition to increased levels.

## Data Availability

Raw data are available for collaboration purposes upon request at corresponding author.
